# Preoperative neutrophil-lymphocyte ratio can significantly predict mortality outcomes in patients with non-muscle invasive bladder cancer undergoing transurethral resection of bladder tumor

**DOI:** 10.18632/oncotarget.14179

**Published:** 2016-12-26

**Authors:** Minyong Kang, Chang Wook Jeong, Cheol Kwak, Hyeon Hoe Kim, Ja Hyeon Ku

**Affiliations:** ^1^ Department of Urology, Seoul National University Hospital, Seoul, Republic of Korea

**Keywords:** urothelial carcinoma, urinary bladder, systemic inflammatory response, neutrophil-lymphocyte ratio, predictors

## Abstract

The prognostic role of systemic inflammatory response (SIR) markers is unclear in patients with non-muscle invasive bladder cancer (NMIBC). Here, we aimed to investigate the prognostic role of various SIR markers in the oncological outcomes in non-muscle invasive bladder cancer (NMIBC) patients at a single institution in Korea. Neutrophil-lymphocyte ratio (NLR), derived-NLR (dNLR), and platelet-lymphocyte ratio (PLR) were examined as SIR markers. We retrospectively collected data of 1,698 NMIBC patients who underwent transurethral resection of the bladder (TURB) between 1990 and 2013. After excluding 147 patients, the study population finally consisted of 1,551 individuals. Overall survival (OS), cancer-specific survival (CSS), recurrence-free survival (RFS), and progression-free survival (PFS) were analyzed by using Kaplan-Meier estimates. Multivariate Cox regression model was adopted to identify the predictors of oncological outcomes. Notably, elevated NLR (≥2.0), dNLR (≥1.5) and PLR (≥124) were associated with poor OS outcomes. Patients with increased NLR, but not dNLR and PLR, only had poor CSS estimates compared to those with lower NLR. However, no significant differences were found in RFS and PFS according to the SIR status. In the multivariate Cox regression analysis, elevated NLR was identified as a key predictor of OS [hazard ratio (HR)=1.52, 95% confidence interval (CI)=1.19-1.95], in addition to age (HR=1.07, 95% CI=1.05-1.08), hemoglobin (HR=0.83, 95% CI=0.78-0.88), and high grade tumor (HR=1.88, 95% CI=1.45-1.08). With respect to CSS, increased NLR was also identified as an independent predictor (HR=1.12, 95% CI=1.01-1.25). In summary, our results indicate that NLR can be a very reliable SIR marker for predicting the oncological outcomes, particularly mortality outcomes.

## INTRODUCTION

Bladder cancer is the most common malignant disease among various genitourinary tract cancers, and it is estimated to be the 7th most common malignancy in male individuals worldwide [1]. Most bladder cancers are pathologically diagnosed as urothelial carcinomas (UC). Approximately 75% of the patients with UC of the urinary bladder have non-muscle invasive disease, such as Ta, T1 and carcinoma *in situ* (CIS) at the time of diagnosis [2, 3]. After initial transurethral resection of bladder tumor (TURB) as the treatment of choice for non-muscle invasive bladder cancer (NMIBC) patients, 70% of the patients may experience recurrence with a high 5-year recurrence rate that ranges from 30% to 80%. Also, 20% to 30% of NMIBC patients progress to muscle invasive bladder cancer requiring radical surgery. To improve therapeutic decision making in these patients, it is important to determine the appropriate predictors of recurrence, progression and survival. However, developing biomarkers for accurate risk classification and selection of high risk patient remains a significant challenge.

Considering that the interaction between systemic inflammatory response (SIR) and tumor plays a key role in cancer development and progression, the neutrophil-to-lymphocyte ratio (NLR) measured in the peripheral blood has been identified as a good predictive marker for pathological and oncological outcomes in various types of malignancies [4]. Similarly, other inflammatory cell-based indicators, including derived NLR (dNLR) and platelet-lymphocyte ratio (PLR), have been suggested as potential prognosticators in cancer patients [5, 6]. Although many studies have reported the role of these systemic inflammatory markers in patients with muscle invasive bladder cancer (MIBC) who underwent radical cystectomy, its consistency and significance as prognosticator are still unclear, particularly in NMIBC patients [7–11].

Here, we hypothesized that preoperative status of well-known SIR markers (NLR, dNLR and PLR) can be significant prognostic factors that predict the oncological outcomes in NMIBC patients who underwent TURB, and sought to elucidate the clinical significance of these SIR markers.

## RESULTS

### Clinicopathological characteristics of patients with NMIBC

Table 1 presents the clinicopathological characteristics of 1,551 patients with NMIBC in this study. The median follow-up duration was 52.0 months [interquartile range (IQR): 27.0 – 82.0]. Median age was 65 years (IQR: 57 – 72) and approximately 80% of the patients (n=1,302) were male. Following the initial TURB at our institution, 50% of the patients (n=785) experienced tumor recurrence, while disease progression occurred in 5.5% of the patients (n=85). The rates of all-cause and cancer-specific death were 16.8% (n=261) and 6.1% (n=95), respectively. With respect to the SIR markers, median values were 1.85 for NLR (IQR: 1.34 – 2.60), 1.36 for dNLR (IQR: 0.99 – 2.38) and 113.0 for PLR (IQR: 87.9 – 186.8), respectively.

**Table 1:** Clinicopathological characteristics of 1,551 patients with NMIBC

Variables	
Age (year)	65 (57 – 72)
BMI (kg/cm^2^)	24.1 (22.1 – 26.0)
Sex (N, %)	
Male	1302 (83.9)
Female	249 (16.1)
Blood cell counts	
Hemoglobin (g/dl)	14.2 (13.1 – 15.1)
Neutrophil counts (x10^3^/μl)	3.57 (2.76 – 4.56)
Lymphocyte counts (x10^3^/μl)	1.93 (1.51 – 2.43)
Platelet counts (x10^3^/μl)	220 (185 – 304)
SIR parameters	
NLR	1.85 (1.34 – 2.60)
dNLR	1.36 (0.99 – 2.38)
PLR	113.0 (87.9 – 186.8)
No. of tumors (N, %)	
Tumor type (N, %)	
Primary	1348 (87.0)
Recurred	202 (13.0)
*Missing (n)*	*1*
No. of tumor	
1	832 (53.6)
2 – 7	63 (40.6)
≥ 8	89 (5.7)
Tumor size (N, %)	
< 3 cm	1283 (82.9)
≥ 3 cm	265 (17.1)
*Missing (n)*	*3*
Pathologic T stage (N, %)	
Ta	888 (57.3)
Tis	65 (4.2)
T1	597 (38.5)
*Missing (n)*	*1*
Tumor grade (N, %)	
PUNLMP	52 (3.4)
Low grade	738 (47.8)
High grade	755 (48.9)
*Missing (n)*	*6*
Concomitant CIS	136 (8.8)
IBCG risk classification	
Low risk	427 (27.5)
Intermediate risk	261 (16.8)
High risk	863 (55.6)
Lymphovascular invasion (N, %)	20 (1.3)
Intravesical chemotherapy (N, %)	368 (23.7)
Oncological outcomes (N, %)	
Recurrence (bladder)	734 (47.3)
Recurrence (upper tract)	51 (3.3)
Progression	85 (5.5)
Radical cystectomy	130 (8.4)
Mortality (N, %)	
All-cause	261 (16.8)
Cancer-specific	95 (6.1)
Follow-up duration (mon)	52 (27 – 82)

Abbreviations: NMIBC, non-muscle invasive bladder cancer; BMI, body mass index; SIR, systemic inflammatory response; NLR, neutrophil-to-lymphocyte ratio; dNLR, derived NLR; PLR, platelet-lymphocyte ratio; PUNLMP, papillary urothelial neoplasm of low malignant potential; CIS, carcinoma *in situ*; IBCG, International Bladder Cancer Group.

### Association of serum SIR markers (NLR, dNLR and PLR) and oncological outcomes in the overall population

**Figure 1:**
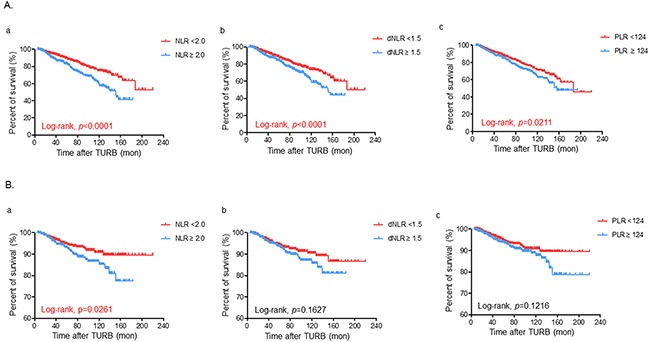
Kaplan-Meier survival estimates for comparing **A**. overall and **B**. cancer specific-survivals according to the preoperative status of (a) neutrophil-lymphocyte ratio (NLR), (b) derived NLR (dNLR), and (c) platelet-lymphocyte ratio (PLR), respectively in the overall population of non-muscle invasive bladder cancer patients who were treated with transurethral resection of the bladder (TURB). Statistical differences were compared between the two groups by using the log-rank test.

We examined whether representative SIR markers (NLR, dNLR and PLR) were associated with various oncological outcomes in the overall population of NMIBC patients using Kaplan-Meier survival analysis. NMIBC patients were classified into two groups according to the preoperative NLR (<2.0 vs ≥2.0), dNLR (<1.5 vs ≥1.5) and PLR values (<124 vs ≥124), respectively. Notably, elevated NLR (≥2.0), dNLR (≥1.5) and PLR (≥124) were significantly associated with poor OS outcomes, as shown in Figure 1A. Patients with elevated NLR, but not dNLR and PLR, only had poor CSS estimates compared to their counterparts (Figure 1B). Interestingly, no significant differences were observed in RFS and PFS rates according to the NLR status (Supplementary Figure 1), as well as other SIR markers (dNLR and PLR) (data not shown).

### Association between serum SIR markers and oncological outcomes in the subgroup population based on risk classification

**Figure 2:**
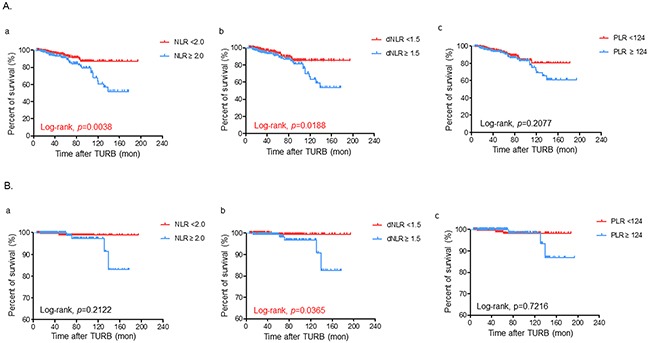
Kaplan-Meier survival curves for comparing **A**. overall and **B**. cancer specific-survivals according to the preoperative status of (a) neutrophil-lymphocyte ratio (NLR), (b) derived NLR (dNLR), and (c) platelet-lymphocyte ratio (PLR), respectively in the low risk population of non-muscle invasive bladder cancer patients based on the risk classification of the International Bladder Cancer Group. Statistical differences were compared between the two groups by using the log-rank test.

**Figure 3:**
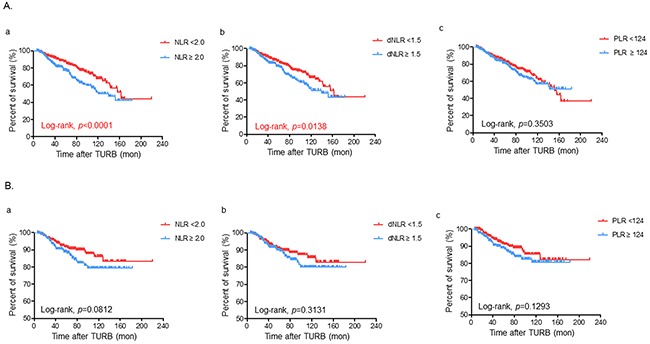
Kaplan-Meier survival curves for comparing **A**. overall and **B**. cancer specific-survivals according to the preoperative status of (a) neutrophil-lymphocyte ratio (NLR), (b) derived NLR (dNLR), and (c) platelet-lymphocyte ratio (PLR), respectively in the high risk population of non-muscle invasive bladder cancer patients based on the risk classification of the International Bladder Cancer Group. Statistical differences were compared between the two groups by using the log-rank test.

We further assessed the prognostic significance of SIR markers in a more specific population stratified by the risk classification of the International Bladder Cancer Group (IBCG). NMIBC patients were also classified into two groups according to the preoperative NLR (<2.0 vs ≥2.0), dNLR (<1.5 vs ≥1.5) and PLR values (<124 vs ≥124), respectively, in each subgroup population. Similar to the overall population, higher NLR (≥2.0) and dNLR (≥1.5), but not PLR, were independently associated with worse OS estimates in both low and high risk patients (Figure 2A and 3A, respectively). However, patients with an elevated dNLR only had a poorer CSS rate compared to those with decreased dNLR, particularly in the low risk population (Figure 2B and 3B). As shown in Supplementary Figure 2, patients with an intermediate risk had comparable oncological outcomes according to any of the SIR markers.

### Identification of significant predictors of overall and cancer-specific survival by the multivariate analysis in the overall population

Furthermore, we performed multivariate Cox regression analysis to determine the predictors of oncological outcomes in patients with NMIBC undergoing TURB. Among various significant variables under the multivariate analysis, elevated NLR (≥2.0) was the key predictive factor for OS (HR=1.52; 95% CI=1.19–1.95), in addition to age (HR=1.07; 95% CI=1.05–1.08), hemoglobin (HR=0.83; 95% CI=0.78–0.88), and high grade tumor (HR=1.88; 95% CI=1.45–2.43) (Table 2). Also, increased NLR (≥2.0) was identified as an independent predictor for CSS (HR=1.12; 95% CI=1.01 – 1.25), indicating that a one-unit increase in NLR would elevate the risk of cancer-specific mortality by 12% in NMIBC patients (Table 3). Based on these results, we compared the clinicopathological features according to the preoperative NLR status (≥2.0 versus <2.0) as the key predictor for survival outcomes, and summarized in Supplementary Table 1.

**Table 2:** Multivariate Cox proportional hazard ratio analysis to identify the predictive factors for overall survival after transurethral resection of bladder tumor

Variables	Univariate			Multivariate		
	HR	95% CI	*P*	HR	95% CI	*P*
**Age (year)**	1.08	1.06 – 1.09	<0.001	**1.07**	1.05 – 1.08	**<0.001**
**Hemoglobin (g/dl)**	0.78	0.75 – 0.83	<0.001	**0.83**	0.78 – 0.88	**<0.001**
**NLR**						
< 2.0	Reference			Reference		
**≥ 2.0**	1.80	1.40 – 2.31	<0.001	**1.52**	1.19 – 1.95	**0.001**
dNLR						
< 1.5	Reference			Reference		
≥ 1.5	1.53	1.19 – 1.95	0.001	0.98	0.72 – 1.32	0.875
PLR						
< 124	Reference			Reference		
≥ 124	1.32	1.04 – 1.69	0.022	0.99	0.76 – 1.31	0.987
Pathologic T stage						
≤ Tis	Reference			Reference		
T1	1.78	1.40 – 2.27	<0.001	1.14	0.85 – 1.52	0.374
**Tumor grade**						
**≤ Low grade**	Reference			Reference		
**High grade**	2.14	1.67 – 2.76	<0.001	**1.88**	1.45 – 2.43	**<0.001**
Lymphovascular invasion						
Absent	Reference			Reference		
Presence	2.81	1.45 – 5.47	0.002	1.48	0.74 – 2.96	0.261

Abbreviations: NLR, neutrophil-to-lymphocyte ratio; dNLR, derived NLR; PLR, platelet-lymphocyte ratio.

**Table 3:** Multivariate Cox proportional hazard ratio analysis to identify the predictive factors for cancer-specific survival after transurethral resection of bladder tumor

Variables	Univariate			Multivariate		
	HR	95% CI	*P*	HR	95% CI	*P*
**Age (year)**	1.06	1.04 – 1.08	<0.001	**1.05**	1.02 – 1.07	**<0.001**
**Hemoglobin (g/dl)**	0.82	0.75 – 0.89	<0.001	**0.88**	0.80 – 0.96	**0.007**
**NLR (continuous)**	1.16	1.06 – 1.28	0.002	**1.12**	1.01 – 1.25	**0.030**
dNLR (continuous)	1.24	1.02 – 1.50	0.033	0.70	0.37 – 1.33	0.277
PLR						
< 124	Reference			Reference		
≥ 124	1.61	1.07 – 2.41	0.021	1.22	0.78 – 1.92	0.370
**Tumor status**						
Primary	Reference			Reference		
**Recurred**	1.95	1.20 – 3.17	0.007	**1.83**	**1.10 – 3.03**	**0.020**
**Pathologic T stage**						
≤ Tis	Reference			Reference		
**T1**	2.75	1.82 – 4.17	<0.001	**1.69**	**1.03 – 2.78**	**0.037**
**Tumor grade**						
≤ Low grade	Reference			Reference		
**High grade**	3.79	2.39 – 6.01	<0.001	**2.21**	**1.28 – 3.82**	**0.004**
Concomitant CIS						
Absent	Reference			Reference		
Presence	2.38	1.32 – 4.30	0.004	1.85	0.96 – 3.55	0.063
**Lymphovascular invasion**						
Absent	Reference			Reference		
**Presence**	6.28	2.91 – 13.58	<0.001	**2.97**	**1.32 – 6.73**	**0.009**

Abbreviations: NLR, neutrophil-to-lymphocyte ratio; dNLR, derived NLR; PLR, platelet-lymphocyte ratio; CIS, carcinoma *in situ*.

### Identification of significant predictors of overall and cancer-specific survivals by the multivariate analysis in the subgroup population based on risk classification

We finally identified the prognosticators of oncological outcomes in the subgroup population divided by the IBCG risk classification (Table 4). Because the univariate analysis revealed that there were no significant variables in the intermediate risk patients, we only analyzed the low and high risk patients. Of note, age (HR=1.14; 95% CI=1.09–1.18) and higher NLR (≥2.0) (HR=2.46; 95% CI=1.34–4.50) were identified as the significant predictors of OS in low risk patients. In high risk patients, elevated NLR (≥2.0) was predictive for OS (HR=1.54; 95% CI=1.14–2.07), in addition to age (HR=1.06; 95% CI=1.04–1.08), hemoglobin (HR=0.82; 95% CI=0.76–0.87) and high grade tumor (HR=1.72; 95% CI=1.09–2.71), after adjusting for other confounding factors. Interestingly, the HR of higher NLR (≥2.0) was more prominent in the low risk population compared to the high risk population (HR=2.46 vs. 1.54).

**Table 4:** Multivariate Cox proportional hazard ratio analysis to identify the predictive factors for overall survival in *low* and *high risk* patients with NMIBC

Variables	Univariate			Multivariate		
	HR	95% CI	*P*	HR	95% CI	*P*
***Low risk patients***						
**Age (year)**	1.13	1.09 – 1.68	<0.001	**1.14**	1.09 – 1.18	**<0.001**
Hemoglobin (g/dl)	0.83	0.74 – 0.93	0.002	0.90	0.79 – 1.03	0.150
**NLR**						
< 2.0	Reference			Reference		
**≥ 2.0**	2.37	1.31 – 4.30	0.004	**2.46**	**1.34 – 4.50**	**0.004**
dNLR						
< 1.5	Reference			Reference		
≥ 1.5	2.01	1.12 – 3.59	0.019	1.19	0.35 – 4.05	0.771
***High risk patients***						
**Age (year)**	1.07	1.05 – 1.08	<0.001	**1.06**	1.04 – 1.08	**<0.001**
**Hemoglobin (g/dl)**	0.79	0.75 – 0.84	<0.001	**0.82**	0.76 – 0.87	**<0.001**
**NLR**						
< 2.0	Reference			Reference		
**≥ 2.0**	1.67	1.24 – 2.25	0.001	**1.54**	1.14 – 2.07	**0.005**
dNLR						
< 1.5	Reference			Reference		
≥ 1.5	1.44	1.07 – 1.94	0.015	0.59	0.32 – 1.09	0.097
**Tumor grade**						
≤ Low grade	Reference			Reference		
**High grade**	1.68	1.07 – 2.64	0.025	**1.72**	1.09 – 2.71	**0.020**
Lymphovascular invasion						
Absent	Reference			Reference		
Presence	2.13	1.09 – 4.18	0.027	1.48	0.74 – 2.98	0.266

Abbreviations: NLR, neutrophil-to-lymphocyte ratio; dNLR, derived NLR.

Additionally, ROC curve analysis showed that NLR had a predictive power to discriminate for OS (AUC=0.601; 95% CI=0.56–0.64; p <0.001) in the overall population (Supplementary Figure 3), as well as the subgroup population according to the risk classification (Supplementary Figure 4). Interestingly, AUC value in the patients with intermediate risk was higher than those with low and high risk patients (0.630 vs. 0.616 and 0.583, respectively).

## DISCUSSION

A complex network of both local and systemic relationships between the host immune system and neoplastic cells plays an important role in tumor growth and progression [12]. Biological characteristics of the tumor and host inflammatory reaction are significantly associated with the prognosis of cancer patients [13]. Therefore, many researchers have studied the clinical significance of SIR markers in cancer patients, because cell-based serum biomarkers, such as NLR, can be efficient biomarkers for simply representing the tumor-host interaction [14–17]. Indeed, an elevated NLR has been shown to be associated with advanced stages and a poor prognosis in variety of human malignancies, such as gastric, colorectal and ovarian cancers [18]. Although NLR can also be a potential prognostic marker for bladder cancer patients, only a small number of reports on this topic in NMIBC patients have been published [19–22]. To the best of our knowledge, this is the first study to highlight the prognostic significance of well-known SIR markers (NLR, dNLR and PLR) in NMIBC patients who underwent TURB. Of note, we showed that among these SIR markers, the preoperative NLR was a strong predictor of OS and CSS in NMIBC patients by using the largest patient population to date.

With accumulating evidence supporting the prognostic values of SIR-based biomarkers, recent studies showed promising results on the prognostic role of SIR markers in bladder cancer patients [15]. Morizawa and colleagues evaluated 110 bladder cancer patients undergoing radical cystectomy, and they observed that higher NLR (≥2.6) was independently associated with RFS (HR=2.61), CSS (HR=2.58), and OS (HR=2.77), respectively [9]. Kawahara *et al*. [10] also demonstrated that bladder cancer patients with a higher NLR (≥2.38) were at a higher risk of cancer-specific death (HR=4.84). Furthermore, a recent first meta-analysis of 23 studies, including 6240 patients’ data, revealed that an increased NLR was significantly correlated with poor cancer-specific (100%), overall (71%), and recurrence-free survival (100%) [23]. Moreover, as our study showed that NLR was the only predictor for oncological outcomes, Bhindi *et al*. [24] reported that NLR was finally identified as the most efficient marker for predicting RFS, CSS and OS, after examining nine cell-based biomarkers based on pre-treatment tests in 418 patients underwent radical cystectomy for bladder cancer. However, these reports primarily focused on the patients with MIBC who underwent radical cystectomy with lymph node dissection.

In NMIBC patients, Kaynar and colleagues revealed a positive correlation of NLR with tumor size (r=0.193) and tumor invasiveness (r=0.138), indicating the prognostic significance of preoperative NLR in this specific population [22]. Moreover, Mano *et al*. [19] recently reported that NLR was an independent predictive factor for tumor recurrence (HR 1.75; 95% CI 1.05-2.92) and disease progression (HR 3.52; 95% CI 1.33-9.33) in 122 consecutive NMIBC patients. Ozalvacli *et al*. [20] also showed that a high pretreatment NLR (≥2.43) was significantly associated with high recurrence rates (HR 2.58; 95% CI 1.15-5.78) in 166 patients with high grade T1 bladder tumors. Interestingly, previous studies on NMIBC patients suggested that pretreatment NLR was primarily associated with the tumor biology-related outcomes (invasiveness, recurrence and progression), while our study showed that preoperative NLR was significantly associated with host condition-related outcomes (mortality outcomes), and not tumor biology-related outcomes (recurrence or progression). In the mechanistic view, the results of Gostas and Kaynar support our findings that an advanced stage and aggressiveness are required for an increased host inflammatory response [25]. Therefore, NLR cannot accurately predict the oncological outcomes, particularly tumor biology-related outcomes, in patients with a lower tumor burden, as in NMIBC. The authors speculated that other comorbidity factors can have more influence on the systemic inflammatory status, making NLR a fragile marker in these patients.

Nevertheless, there is a critical scientific rationale for adopting NLR in cancer patients, particularly in the context of tumor-host interactions. NLR indicates the relative ratio of the neutrophil count as the innate immune reaction to the lymphocyte count as the adaptive immune activity. Since the Virchow's hypothesis was proposed in 1863, the concept of a complex interplay network between inflammatory reaction, innate immunity and cancer has been more widely accepted over the last decade [26]. Cancer cells produce various cytokines, such as tumor necrosis factor-α and interleukin-6, and this phenomena can increase the number of tumor-infiltrating neutrophils around a tumor [27]. Increased number of neutrophils (neutrophilia) can induce DNA damage by secreting reactive oxygen species, stimulating the proliferation of cancer cells, and promoting neo-vascularization around cancer tissues [28]. Conversely, tumor-infiltrating lymphocytes can recognize the tumor cells, and finally induce apoptotic cell death through direct or indirect cytotoxicity [29]. Thus, lymphopenia may represent reduced cytotoxic T cell activities and attenuation of the anti-cancer immune responses.

We should consider the critical drawbacks of the current study. First of all, this is a retrospective study performed at a single tertiary center, which has unavoidable selection biases. Second, single preoperative tests for SIR markers cannot fully represent the dynamics of systemic inflammatory activities. Third, although we only explored the role of cell-based SIR markers due to the retrospective nature of this study, acute phase protein-based markers, such as C-reactive protein and albumin, should be required for obtaining more concrete evidence for the clinical utility of SIR markers in NMIBC patients. Fourth, although the cut-off values of each SIR markers were selected using ROC curve analysis, our data showed lower cut-off values compared to other studies. We speculate that differences in host immune responses against tumors, individual's disease status, ethnicity, performance status, and time points of laboratory tests for SIR markers can significantly affect the preoperative status of these markers. Finally, there were no supportive data from the mechanistic view, such as the quantitative histologic scoring of tumor-infiltrating immune cells around the tumor.

In conclusion, we found that there was a significant association between higher NLR and OS and CSS, but not between higher NLR and PFS and RFS, in NMIBC patients who underwent TURB by evaluating well-known SIR markers (NLR, dNLR and PLR). Our results suggest that NLR is the most valuable SIR marker for predicting the oncological outcomes, particularly the mortality outcomes, and guiding the appropriate clinical decision-making in NMIBC patients.

## MATERIALS AND METHODS

### Study sample

The Institutional Review Board at our center approved the current study (H-1606-029-768). Because our study was performed retrospectively, the requirement for obtaining informed consent from the patients was waived. All study protocols including human resources were based on the Declaration of Helsinki guidelines.

We initially reviewed the medical records of 1,698 patients with NMIBC who underwent TURB from March 1990 to December 2013 at Seoul National University Hospital. We excluded the patients with a non-urothelial carcinoma histology, a short-term follow-up period of less than six months following initial TURB, and clinical evidence of systemic inflammation, such as fever and leukocytosis. We also excluded the patients with incomplete clinical and pathological information, particularly the serum parameters representing SIR. Our study population finally consisted of 1,551 NMIBC patients who were included in the analysis.

### Study design

For accurate diagnosis and treatment of NMIBC patients, TURB was performed according to the standard surgical steps established at our hospital. Resected tumor specimens were specifically assessed by histological examination, and reviewed by two experienced genitourinary pathologists. The 2010 American Joint Committee on Cancer classification and the 2004 World Health Organization/International Society of Urologic Pathology consensus classifications were adopted to determine the TNM stage and tumor grade, respectively. The preoperative laboratory measurements, including a complete blood count test, were routinely carried out within one month before TURB.

We collected clinicopathological information on NMIBC patients in terms of the following parameters: age at initial TURB performed at our hospital, gender, body mass index (BMI), hemoglobin (g/dl), absolute neutrophil, lymphocyte and platelet counts (/μl), preoperative NLR, dNLR and PLR values, the type of tumor (primary or recurred tumor), the number and size of the tumors, pathological T stage, tumor grade, the presence of carcinoma *in situ* and lymphovascular invasion (LVI), and various oncological outcomes such as initial recurrence, progression, cancer-specific mortality and all-cause mortality.

The NLR and dNLR were calculated using the following formulas: NLR = absolute neutrophil count/lymphocyte count; dNLR = absolute neutrophil count/ (white blood cell count – neutrophil count). PLR was calculated as follows: the ratio of absolute platelet count to lymphocyte count. We used the receiver-operating characteristic curve analysis in order to determine the appropriate cut-off points for these SIR markers, respectively, as described elsewhere [30]. The optimal cut-off values were chosen as they appeared to maximize the sensitivity and specificity for predicting oncological outcomes, which had the maximal value of Youden index [30].

NMIBC patients were monitored every three months for the first two years after the initial TURB. Follow-up examinations after surgery consisted of history taking, physical examination, routine laboratory tests, urine cytology and cystoscopic examination. The patients were followed up every six months for three to four years after the initial treatment, and then annually. Computed tomography scan was conducted every year to evaluate the status of the upper urinary tract in addition to the routine surveillance.

### Statistical analysis

For describing the outcomes of statistical analysis, continuous variables were presented as the median values with an interquartile range (IQR) and categorical variables were demonstrated as the proportion of events (%). The two-sided *p*-values less than 0.05 were considered to indicate a statistically significant status. IBM SPSS Statistics version 22.0 (IBM, Armonk, New York, USA) and GraphPad Prism version 5.0 (GraphPad Software Inc., San Diego, CA, USA) were used for all statistical tests in the current study.

Among various oncological outcomes, overall survival (OS) estimate was the primary end point of this study. With respect to the secondary end points, recurrence-free survival (RFS), progression-free survival (PFS) and cancer-specific survival (CSS) were analyzed. Specific survival was estimated from the time of the initial TURB until the occurrence of relevant events. If there was no specific event, patients were treated as having the censored state at the last follow-up. The Kaplan-Meier survival analysis was adopted to calculate all survival outcomes, and the log-rank test was used to compare the relevant oncological outcomes between the two groups. Finally, Cox proportional hazard regression analysis was used to determine the independent predictors of various oncological outcomes. In order to avoid the unexpected confounding effects of other variables, only those factors that were statistically significant in the univariate analysis were included in the multivariate analysis by applying the backward stepwise procedure.

## SUPPLEMENTARY MATERIALS FIGURES AND TABLES




