# Supplementary material for: Preoperative neutrophil-lymphocyte ratio can significantly predict mortality outcomes in patients with non-muscle invasive bladder cancer undergoing transurethral resection of bladder tumor
Source: Oncotarget. 2016 Dec 26;8(8):12891–901. doi: 10.18632/oncotarget.14179 (PMC5355064; doi:10.18632/oncotarget.14179)
Supplement: Supplementary file 1 [file oncotarget-08-12891-s001.pdf]

Preoperative neutrophil-lymphocyte ratio can significantly predict mortality outcomes in patients with non-muscle invasive bladder cancer undergoing transurethral resection of bladder tumor

SUPPLEMENTARY FIGURES AND TABLE

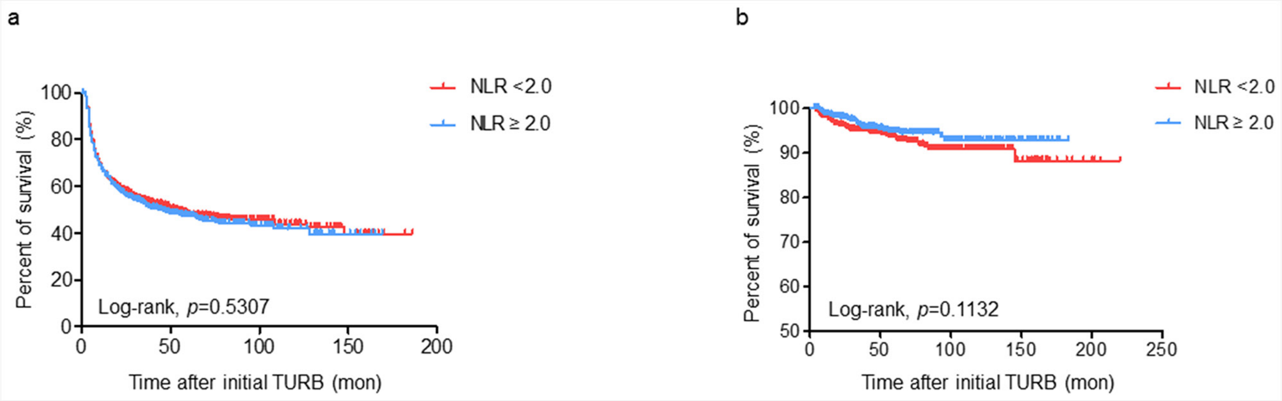

Supplementary Figure 1: Kaplan-Meier survival estimates for comparing **a.** intravesical recurrence-free survival and, **b.** progression-free survival according to the preoperative neutrophil-lymphocyte ratio (NLR) status in the overall population of non-muscle invasive bladder cancer patients, who were treated with transurethral resection of the bladder (TURB). Statistical differences were compared between the two groups by using the log-rank test.

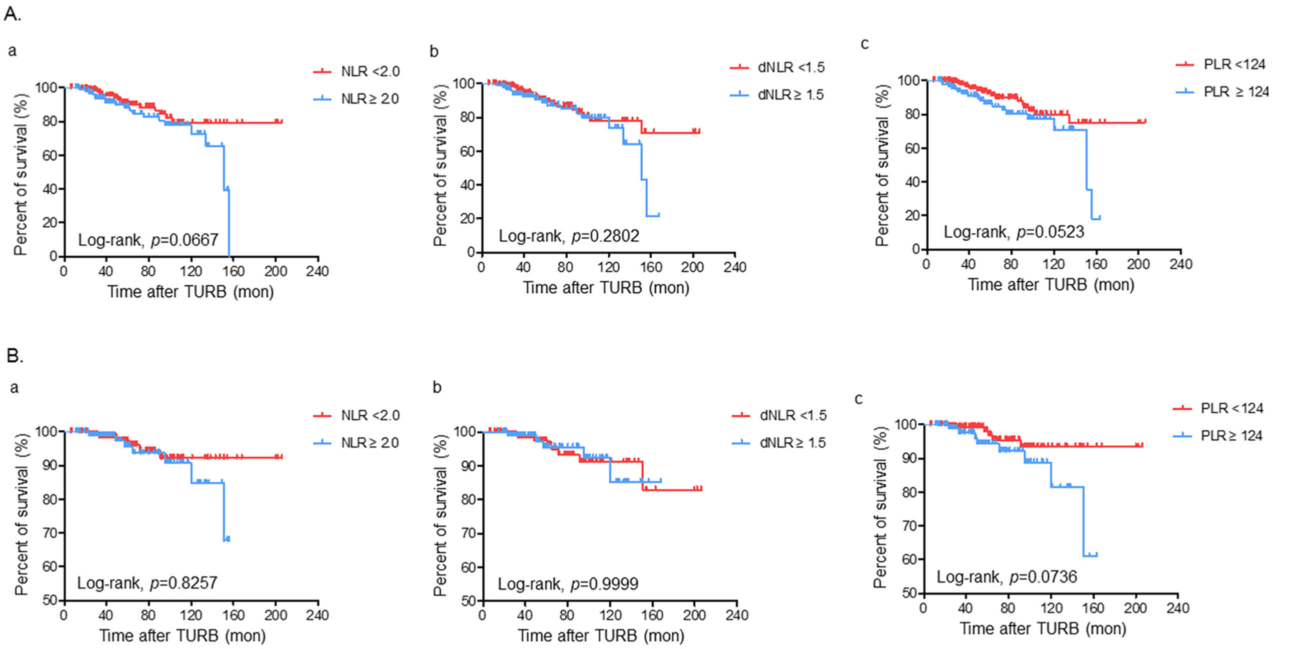

Supplementary Figure 2: Kaplan-Meier survival curves for comparing A. overall and **B.** cancer specific-survivals according to the preoperative status of (a) neutrophil-lymphocyte ratio (NLR), (b) derived NLR (dNLR), and (c) platelet-lymphocyte ratio (PLR), respectively in the intermediate risk population of non-muscle invasive bladder cancer patients based on the risk classification of the International Bladder Cancer Group. Statistical differences were compared between the two groups by using the log-rank test.

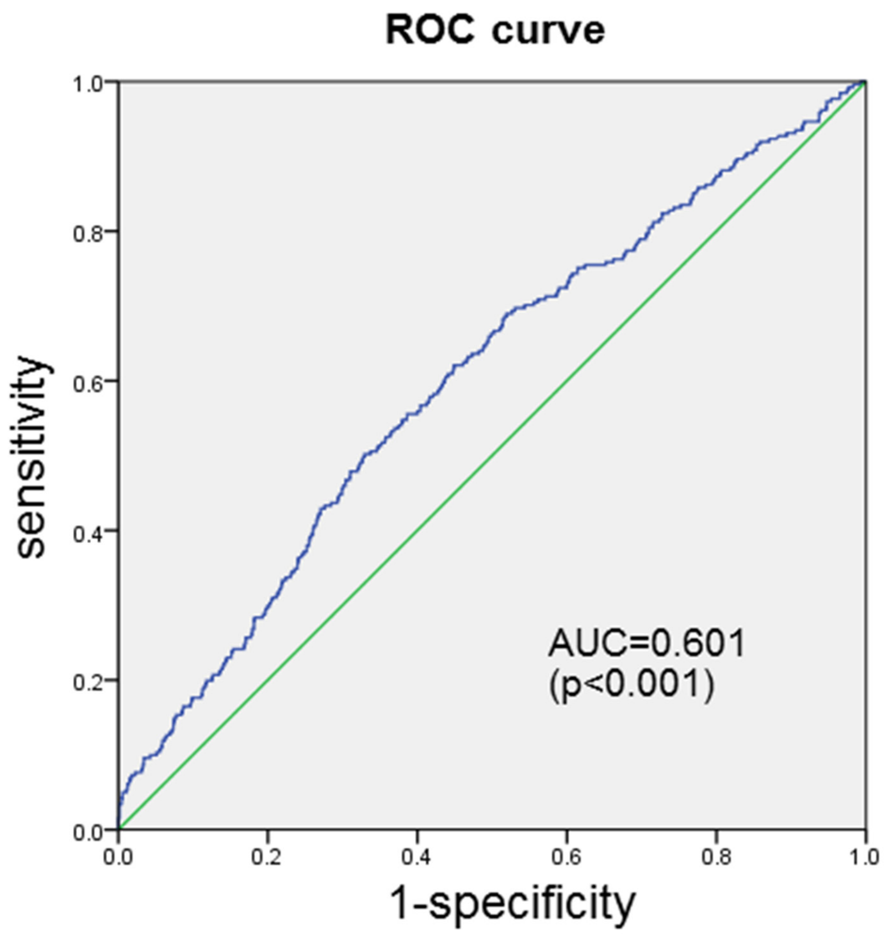

Supplementary Figure 3: Receiver operating characteristic curve analysis of the ability of neutrophil-lymphocyte ratio to discriminate for overall survival in overall population of non-muscle invasive bladder cancer patients.

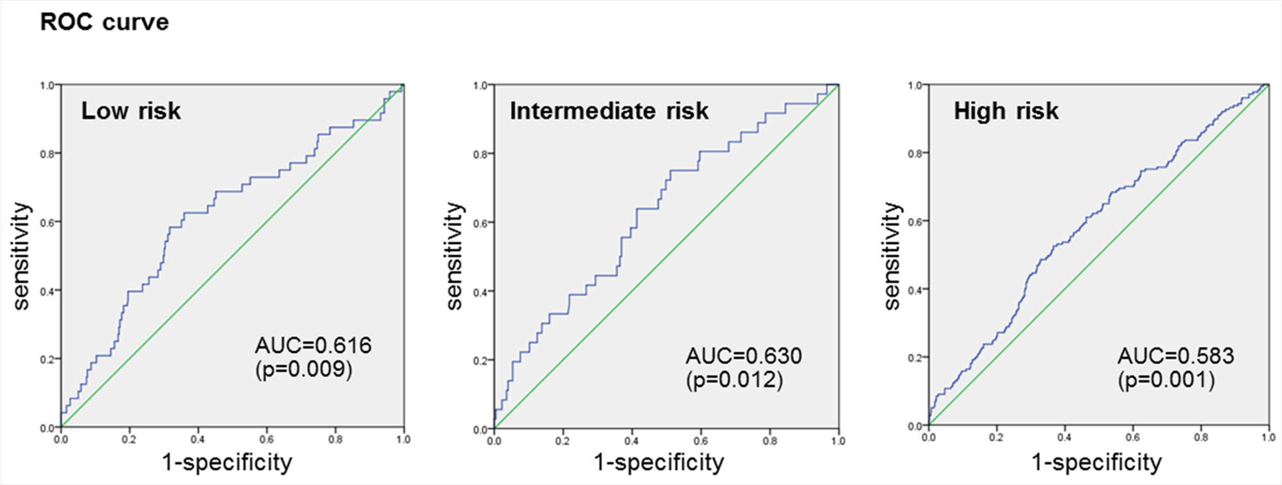

Supplementary Figure 4: Receiver operating characteristic curve analysis for the ability of neutrophil-lymphocyte ratio to discriminate for overall survival in subgroup population of non-muscle invasive bladder cancer patients according to the International Bladder Cancer Group risk classification.

Supplementary Table 1: Comparing the clinicopathological features according to the preoperative NLR status in patients with NMIBC.

See Supplementary File 1
