# Supplementary material for: Preoperative neutrophil-lymphocyte ratio can significantly predict mortality outcomes in patients with non-muscle invasive bladder cancer undergoing transurethral resection of bladder tumor
Source: Oncotarget. 2016 Dec 26;8(8):12891–901. doi: 10.18632/oncotarget.14179 (PMC5355064; doi:10.18632/oncotarget.14179)
Supplement: Supplementary file 2 [file oncotarget-08-12891-s002.doc]

**Table S1. Comparing the clinicopathological features according to the preoperative NLR status in patients with NMIBC**

| **Variables** | **NLR <2.0**  **(n = 881)** | **NLR ≥2.0**  **(n = 670)** | ***P* value** |
| --- | --- | --- | --- |
| Age (year) | 64 (56 – 58) | 67 (58 – 74) | <0.001 |
| BMI (kg/cm^2^) | 24.3 (22.5 – 26.2) | 23.8 (21.7 – 25.6) | <0.001 |
| Sex (N, %) |  |  |  |
| Male | 723 (82.1) | 579 (86.4) | 0.021 |
| Female | 158 (17.9) | 91 (13.6) |  |
| Blood cell counts |  |  |  |
| Hemoglobin (g/dl) | 14.2 (13.2 – 15.2) | 14.1 (12.8 – 15.1) | 0.009 |
| Neutrophil counts (x10^3^/μl) | 3.04 (2.39 – 3.75) | 4.50 (3.65 – 5.51) | <0.001 |
| Lymphocyte counts (x10^3^/μl) | 2.27 (1.85 – 2.75) | 1.55 (1.28 – 1.87) | <0.001 |
| Platelet counts (x10^3^/μl) | 220 (187 – 263) | 219 (182 – 260) | 0.613 |
|  |  |  |  |
| SIR parameters |  |  |  |
| NLR | 1.41 (1.09 – 1.68) | 2.75 (2.31 – 4.50) | <0.001 |
| dNLR | 1.06 (0.85 – 1.27) | 1.93 (1.67 – 2.31) | <0.001 |
| PLR | 97.3 (75.9 – 121.5) | 137.3 (112.4 – 175.7) | <0.001 |
| Tumor type (N, %) |  |  |  |
| Primary | 755 (85.8) | 593 (88.5) | 0.128 |
| Recurred | 125 (14.2) | 77 (11.5) |  |
| *Missing (n)* | *1* | *0* |  |
| No. of tumor |  |  |  |
| 1 | 465 (52.8) | 367 (54.8) | 0.434 |
| 2 – 7 | 360 (40.9) | 270 (40.3) |  |
| ≥ 8 | 56 (6.4) | 33 (4.9) |
| Tumor size (N, %) |  |  |  |
| < 3 cm | 740 (84.1) | 543 (81.3) | 0.177 |
| ≥ 3 cm | 140 (15.9) | 125 (18.7) |  |
| *Missing (n)* | *1* | *2* |  |
| Pathologic T stage (N, %) |  |  |  |
| Ta | 507 (57.6) | 381 (56.9) |  |
| Tis | 40 (4.5) | 25(3.7) |  |
| T1 | 333 (37.8) | 264 (39.4) |
| *Missing (n)* | *1* | *0* |  |
| Tumor grade (N, %) |  |  |  |
| PUNLMP | 31 (3.5) | 21 (3.1) | 0.571 |
| Low grade | 428 (48.7) | 310 (46.5) |
| High grade | 419 (47.7) | 336 (50.4) |
| *Missing (n)* | *3* | *3* |  |
| Concomitant CIS | 79 (9.0) | 57 (8.5) | 0.751 |
| IBCG risk classification |  |  |  |
| Low risk | 246 (27.9) | 181 (27.0) | 0.518 |
| Intermediate risk | 155 (17.6) | 106 (15.8) |
| High risk | 480 (54.5) | 383 (57.2) |
| Lymphovascular invasion (N, %) | 7 (0.8) | 13 (1.9) | 0.048 |
| Intravesical chemotherapy (N, %) | 203 (23.0) | 165 (24.6) | 0.467 |
| Oncological outcomes (N, %) |  |  |  |
| Recurrence (bladder) | 413 (46.9) | 321 (47.9) | 0.687 |
| Recurrence (upper tract) | 31 (3.5) | 20 (3.0) | 0.667 |
| Progression | 56 (6.4) | 29 (4.3) | 0.091 |
| Radical cystectomy | 68 (7.7) | 62 (9.3) | 0.309 |
| Mortality (N, %) |  |  |  |
| All-cause | 113 (12.8) | 148 (22.1) | <0.001 |
| Cancer-specific | 44 (5.0) | 51 (7.6) | 0.042 |
| Follow-up duration (mon) | 55 (26 – 83) | 49 (27 – 80) | 0.184 |

**Abbreviations**: NLR, neutrophil-to-lymphocyte ratio; NMIBC, non-muscle invasive bladder cancer; BMI, body mass index; SIR, systemic inflammatory response; dNLR, derived NLR; PLR, platelet-lymphocyte ratio; PUNLMP, papillary urothelial neoplasm of low malignant potential; CIS, carcinoma *in situ*; IBCG, International Bladder Cancer Group
